# Laparoscopic-assisted full-sized liver transplantation with magnetically fast portal vein anastomosis: an initial cohort study

**DOI:** 10.1097/JS9.0000000000001730

**Published:** 2024-05-29

**Authors:** Xue-Min Liu, Yu Li, Zhe Feng, Xiao-Gang Zhang, Shan-Pei Wang, Jun-Xi Xiang, Si-Nan Liu, Kun Guo, Jing-Jing Hou, Ai-Hua Shi, Xu-Feng Zhang, Yi Lyu

**Affiliations:** a Department of Hepatobiliary Surgery and Institute of Advanced Surgical Technology and Engineering, The First Affiliated Hospital of Xi'an Jiaotong University; b Department of Surgical Intensive Care Unit, The First Affiliated Hospital of Xi’an Jiaotong University; c National Local Joint Engineering Research Center for Precision Surgery and Regenerative Medicine, Xi’an Jiaotong University, Xi’an, People’s Republic of China

**Keywords:** laparoscopy, liver transplant, magnetic anastomosis

## Abstract

**Background:**

Some cases of laparoscopic-assisted liver transplantation (LA-LT) with utilization of reduced-size grafts has been reported. The authors here introduced successful utilization of LA-LT with whole liver grafts and magnetic portal vein anastomosis.

**Methods:**

Eight patients with liver cirrhosis were included for LA-LT using donor organs after cardiac death. The surgical procedures included purely laparoscopic explant hepatectomy and whole-liver graft implantation via the midline incision. After explant removal, the whole-liver graft was then placed in situ, and a side-to-side cavo-caval anastomosis with 4–5 cm oval opening was performed. The magnetic rings were everted on the donor and recipient portal vein, respectively, and the instant attachment of the two magnets at the donor and recipient portal vein allowed fast blood reperfusion, followed by continuous suturing on the surface of the magnets.

**Results:**

The median operation time was 495 (range 420–630). The median time of explant hepatectomy and inferior vena cava anastomosis was 239 (range 150–300) min and 14.5 (range 10–19) min, respectively. Of note, the median anhepatic time was 25 (range 20–35) min. All the patients were discharged home with no major complications after more than 12 months follow-up.

**Conclusion:**

LA-LT with full-size graft is feasible and utilization of magnetic anastomosis would further simplify the procedure.

## Introduction

Highlights

- Pure laparoscopic explant liver resection is safe and feasible.
- Cavo-caval anastomosis between the recipient and donor full-sized liver is applicable via midline incision with no stenosis of hepatic outflow during more than 1-year follow-up.
- Magnetic-assisted portal vein anastomosis significantly simplifies the procedure, and shortens the anhepatic time, which might further promote broad application of laparoscopic liver transplantation.

Laparoscopic-assisted liver transplantation (LA-LT) has long been thought as a ‘dream’ due to unsolved issues such as explant hepatectomy and removal, long-time vascular clamping, and complex vessels, and bile duct anastomosis. Dokmak and colleagues^1^ recently reported the first case of LA-LT with pure laparoscopic explant hepatectomy and open implantation with a reduced right lobe via midline incision, followed by another report of LA-LT with reduced graft from Suh *et al*.^2^. In addition, Suh and colleagues^3^ have successfully performed pure laparoscopic/robotic living donor liver transplantation. Although this is a breakthrough in living donor liver transplantation, a total operation time of 17 h, ischemia time of 84 mins and an entire portal vein clamping time of 2 h have challenged the application of this complex technique and qualification of potential candidates. In fact, the previous studies introduced LA-LT with reduced rather than whole-liver graft, as laparoscopic vascular anastomosis must be most difficult with full graft implantation. The author’s group has worked on magnetic vascular and digestive tract anastomosis for 20 years^4–13^, and have successfully utilized magnetic-assisted vascular anastomosis in open liver transplantation with a median anhepatic time of 10.5 mins^7^. Based on our previous animal study of LA-LT with magnetic-assisted vascular anastomosis^6^, and clinical experience with magnetic anastomosis in open liver transplantation^7^, we here introduced successful utilization of LA-LT and a simplified magnetic-assisted fast portal vein anastomosis with whole liver grafts.

## Methods

### Patient selection

Ten patients with liver cirrhosis with or without liver cancer were included between 1 November 2022 to 15 February 2023 for LA-LT. LA-LT was considered among adult patients (≥18 years old) with no previous history of open upper abdominal operations. In addition, the selection criteria included: the performance status ≤1, tumor status fulfilling Hangzhou Criteria of China, and no portal thrombosis. All cases suitable for LA-LT were discussed at a multidisciplinary team meeting, and informed consent were obtained from all the patients and their families. However, conversion to open surgery was performed in two patients due to massive bleeding from the varices, and a total of eight patients were finally enrolled. None of the organs were procured from executed prisoners and the donor organs were procured after informed consent. The study was approved by the institutional ethical committee (XJTU1AF2015LSL-046). The work has been reported in line with the strengthening the reporting of cohort, cross-sectional, and case–control studies in surgery (STROCSS) criteria^14^.

### Surgical procedures

**Figure 1:**
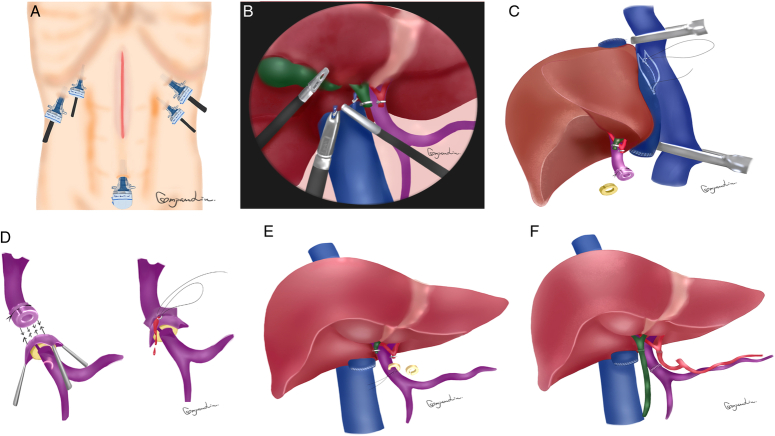
Illustration of laparoscopic-assisted full-sized liver transplantation. (A & B) Laparoscopic explant hepatectomy. (C) Cavo-caval anastomosis via upper abdominal midline incision. (D) Magnetic anastomosis of portal vein and fast restoration of blood flow. (E & F) Removal of the magnets followed by routine artery and bile duct anastomosis.

**Figure 2:**
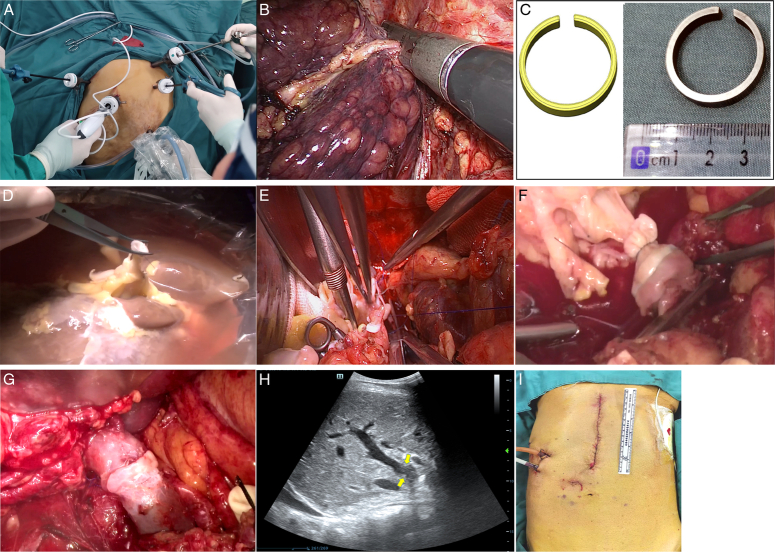
Surgical procedures of laparoscopic-assisted full liver transplantation. (A) Trocar position. (B) Dissection of the right hepatic vein. (C) Magnetic rings used. (D) Installation of the magnetic ring on the donor portal vein. (E) Cavo-caval anastomosis. (F) Attachment of the two magnets on the donor and recipient portal vein, followed by fast restoration of blood flow. (G) Removal of the magnets after safe suture between. (H) Intraoperative ultrasonography confirms patent anastomotic stoma. (I) The final aspect of upper midline incision.

The surgical procedures included purely laparoscopic explant hepatectomy, whole-liver graft implantation, and magnetic-assisted portal vein anastomosis via the midline incision (Supplementary Video, Supplemental Digital Content 1, http://links.lww.com/JS9/C680). Briefly, the recipients were placed in the supine position with the legs apart. Five trocars were used as illustrated (Figs 1A, 2A). The recipient’s liver was mobilized with dissection of the ligaments around. Cholecystectomy was performed and followed by dissection of the common bile duct. In addition, the left and right hepatic artery were dissected, and the portal vein was ready for dissection at the end of the explant removal. The short hepatic veins were clipped and dissected for freeing the caudate lobe from the inferior vena cava (IVC). The portal vein, the common trunk of the left and middle hepatic veins, as well as the right hepatic vein were sequentially divided using an endovascular 45 mm stapler as long as the graft was ready with the magnetic rings fixed on the stump of the portal vein (Figs 1B, 2B, C). On the back table, the C-shaped magnetic ring was placed on portal vein of the donor liver, and the vascular wall was everted on the magnet and ligated for fixation with the gap of the magnetic ring at 12 o’clock direction for prevention of vascular distortion (Fig. 2D). The diameter of the magnetic rings should be equal or 1 mm smaller than the diameter of the donor portal vein for successful eversion. Typically, a pair of magnetic rings 8 or 9 mm in diameter were used for both the donor and recipient portal vein.

The explant native liver was then removed from the upper abdominal midline incision (15–20 cm). The supra-hepatic and infra-hepatic IVC was occluded. The whole-liver graft was then placed in situ, and a side-to-side anastomosis of the donor and recipient IVC with 4–5 cm oval opening was performed (Fig. 1C, 2E, Supplementary video, Supplemental Digital Content 1, http://links.lww.com/JS9/C680). After clamping of the recipient portal vein with nontraumatic forcep, the other C-shaped magnetic ring was similarly placed on the vessel, and the vascular wall was everted on the magnet by three forceps at an appropriated position with the gap of the magnetic ring at 12 o’clock direction as well for prevention of vascular distortion. The two magnets at the stumps of the donor and recipient portal vein attached immediately for a fast portal blood restoration after removal of occlusion forcep on the recipient portal vein and ligation of the donor portal vein (Fig. 1D, 2F). And then the forceps on the IVC was removed as well for complete restoration of the portal and systematic blood flow. The gaps of the two magnets allowed bloodletting, and continuous suture of the portal vein was started at the gaps on the two magnets under the circumstance of blood restoration (Fig. 1D). After suture, the two C-shaped magnets were then removed, and the suture was knotted with a regular growth factor remained (Fig. 1E, 2G). The hepatic artery and bile ducts were anastomosed regularly (Fig. 1F). Intraoperative ultrasound was routinely used for detection of the patency of the portal vein and hepatic artery (Fig. 2H). Finally, the midline incision of 15–20 cm long was closed (Fig. 2I).

### Postoperative care

All the patients received a standard triple immunosuppression regimen in our department including calcineurin inhibitors (Tacrolimus), steroids, and mycophenolate mofetil. The blood concentration of tacrolimus was maintained at 6–8 ng/ml in most patients, and 4–6 ng/ml among cancer patients. Doppler ultrasounds were routinely performed every day for monitoring the patency of the anastomosed vascular, as well as the fluid collection in the chest and abdominal cavity. Even after discharge, the patients were regularly followed up in the Outpatient Center every 2 weeks within the first 3 months, and every month thereafter.

## Results

A total of eight patients (six males and two females) were finally selected for LA-LT (Table 1). The median age was 46 years [interquartile range (IQR) 27.3–54.3], and the median BMI was 22.4 (IQR 18.4–25.7). The primary underlying disease included hepatitis B-associated liver cirrhosis in 4, hepatitis C-associated liver cirrhosis in 1, alcoholic liver disease in 1, and autoimmune hepatitis (AIH) in two patients. Among them, five patients developed hepatocellular carcinoma (HCC) associated with hepatitis B/C or AIH. The median total operation time of the recipients were 495 (range 420–630) min. Specifically, the median time to explant hepatectomy and IVC anastomosis was 239 (range 150–300) min and 14.5 (range 10–19) min, respectively. Of note, the median anhepatic time was 25 (range 20–35) min (Table 2). The median hospital stay was 23 (range 14–32) days. Only one patient had developed acute rejection in hospital and recovered well after treatment with methylprednisolone. All the patients were discharged home with no delayed complications or tumor recurrence at the end of follow-up (April 2024).

**Table 1:** Baseline characteristics and surgical information of the patients undergoing laparoscopically assisted whole liver transplantation.

Demographic	Case 1	Case 2	Case 3	Case 4	Case 5	Case 6	Case 7	Case 8
Age (years)	44	52	66	48	55	34	25	24
Sex	Male	Male	Female	Male	Male	Female	Male	Male
Height (cm)	170	180	163	174	170	157	180	180
Weight (Kg)	52	86	45	75	75	48	80	65
BMI (Kg/m^2^)	18.0	26.5	16.9	24.8	26.0	19.5	24.7	20.1
Underlying liver disease	Hepatitis B	Hepatitis C	AIH	Alcoholic	Hepatitis B	AIH	Hepatitis B	Hepatitis B
Hepatocellular carcinoma	None	Yes	Yes	None	Yes	None	Yes	Yes
Size	–	80×70×45 mm^3^	8×10×12 mm^3^	–	15×12×10 mm^3^	–	50×40×40 mm^3^	50×40×30 mm^3^
Number	–	1	1	–	1	–	1	1
Pre-LT biochemical examination								
Hemoglobin (g/l)	132	121	92	85	145	108	155	167
Platelet (×10^9^/l)	73	48	44	51	112	141	108	157
Total bilirubin (µmol/l)	18.7	81	564.5	192.4	37.6	210	30.4	15
Albumin (g/l)	37.9	29.2	35.6	32.4	34.5	29.2	47.5	44
Prothrombin time (s)	16.8	15.5	19.5	23.1	15	16.2	14.1	12.6
International normalized ratio	1.33	1.42	1.61	1.99	1.24	1.47	1.08	1.12
Alpha-fetoprotein (ng/ml)	4	6268	980	2	9500	10	2565	49.6
Image findings								
Portal thrombosis	–	RPV	–	–	–	–	–	–
Varicosity	EGV	EGV	EGV	EGV	EGV	EGV	EGV	EGV
Child-Pugh score	6	8	9	10	8	10	5	5
MELD score	10	16	20	20	11	20	8	8
Total operation time (min)	450	510	480	420	600	630	555	475
Time to explant hepatectomy (min)	180	250	150	199	300	280	240	238
Cold ischemia time (min)	300	360	300	240	270	300	300	330
Warm ischemia time (min)	72	50	47	50	40	60	48	45
Time of IVC anastomosis (min)	17	15	14	10	14	19	17	14
Anhepatic time (min)	35	25	25	30	20	27	20	25
Time of artery anastomosis (min)	25	22	20	33	31	19	16	21
Time of bile duct anastomosis (min)	15	17	13	21	35	18	16	14
Estimated blood loss (ml)	1000	2500	2600	3000	960	960	150	200
Transfusion, pack								
Red blood cells (U)	4	10	12	18	4	4	0	0
Frozen fresh plasma (ml)	2200	1200	1600	2000	1000	1000	1000	1000
Platelet (U)	0	10	0	0	0	0	0	0
Hospital stay (d)	26	21	28	18	14	32	17	24
Postoperative complication	Acute rejection	No	No	No	No	No	No	No

AIH, autoimmune hepatitis; EGV, esophagogastric varices; LT, liver transplantation; MELD, model for end-stage liver disease; RPV, right portal vein.

**Table 2:** Comparison of surgery-associated parameters and outcomes among laparoscopic/robotic liver transplantation published.

Authors	Dokmak^1^	Lee^18^	Suh^2^	Suh^17^	Suh^3^	Dokmak^15^	Our study
Year	2020	2021	2021	2022	2022	2022	2023
Case number	1	1	3	1	1	6	8
Primary disease	Neuroendocrine liver metastasis	Primary biliary cirrhosis	NA	Alcoholic liver cirrhosis and HCC	Autoimmune hepatitis	Neuroendocrine liver metastasis	HBV/HCV-related liver cirrhosis and/or HCC
Surgery	Right split liver	Reduced-size	Reduced-size	Right liver graft	Right liver graft	Reduced (*n*=3), full (*n*=2), and a right split liver (*n*=1).	All full-sized grafts
Operation time (median, min)	400	740	575	960	1065	405	495
Cold ischemia time (median, min)	466	220	NA	NA	NA	35	300
Warm ischemia time (median, min)	38	87	NA	84	NA	438	49
liver explanation (median, min)	225	140	180	369	260	NA	239
Time of IVC anastomosis (median, min)	NA	35	NA	42	41	NA	14.5
Anhepatic time (median, min)	43	180	161	185	240	51	25
Blood loss (median, ml)	400	3600	NA	3300	11 500	425	980
Recovery	Well	Well	All Well	Well	Well	All well	All well

## Discussion

Inadequate exposure and complex vascular anastomosis are major challenges for LA-LT. Our groups have successfully utilized magnetic anastomosis of IVC and portal vein in open surgery^7^. In the current study, we reported eight successful cases of LA-LT of full-liver graft with utilization of simplified magnetic portal vein anastomosis via upper midline incision. Specifically, the current study demonstrated that pure laparoscopic explant liver resection is safe and applicable. In addition, cavo-caval anastomosis between the recipient and donor full-sized liver is feasible via the midline incision, and no stenosis of hepatic outflow was identified after more than 12-month follow-up postoperatively. Moreover, simplified installation of the magnetic rings on the portal vein of the recipient and donor significantly shortens the anhepatic time, and allows for fast blood restoration of the portal vein. As such, the current study strongly suggests that LA-LT is feasible and safe in appropriately selected patients, and magnetic-assisted vascular anastomosis significantly reduced anhepatic time during full-sized LT, which might further promote broad application of LA-LT.

Laparoscopic explant hepatectomy among patients with liver cirrhosis must be more difficult due to the poor coagulation function and severe varicosity. In fact, liver transplant recipients are typically very sick patients receiving a highly complex life-saving operation. As such, patient selection for LA-LT are important. For consideration of LA-LT, our selection criteria includes (1): adult patients with no actively severe complication such as hepatic encephalopathy, upper gastrointestinal bleeding, or severe splenomegaly beyond the midline; (2) with no obesity (BMI ≥27.5) or previous history of open abdominal surgery; (3) with no thrombosis in the trunk of portal vein. In addition, patients with ascites and small native livers would be better candidates for LA-LT for adequate spaces. And also, a small graft would be more suitable for this procedure because of easier exposure for vascular anastomosis. However, conversion to open surgery is anytime considered in case of occasional catastrophic events occurring, such as uncontrollable major bleeding.

With progression of minimally invasive surgery being predominantly common in operation room, laparoscopic/robotic liver transplantation has been attempted in several high-volume institutions. Specifically, with reported experience of laparoscopic-assisted living-donor liver transplantation via midline incision^1,15^, pure laparoscopic/robotic living donor liver transplantation has been recently reported. However, a total operation time of 16–17 h, warm ischemia time of 84 mins and an entire portal vein clamping time of 2 h have questioned the broad application of this complex technique on very sick candidates^3,16,17^. In addition, one study reported LA-LT among six patients with neuroendocrine liver metastasis using upper midline incision with anhepatic time of 40–67 mins^15^. Up to date, a total of 13 patients who underwent laparoscopic or robotic liver transplantation has been performed before the current study, and most of them are case report (Table 2). A total operation time of 495 min in our cases were similar to other reports with laparoscopic-assisted liver transplantation^1,2,15,18^, but significantly shorter than pure laparoscopic/robotic procedure^3,17^. In fact, all the previous studies mainly reported living-donor liver transplantation with reduced size of grafts. However, in areas such as China, full-sized graft from cadaveric donors are mainly used. Under pure laparoscope or robotics, it is extremely difficult to expose the vascular for anastomosis during full-sized liver transplantation. As such, for full-sized graft implantation, an upper midline incision is appropriate and sufficient for explant removal, graft placement, and vascular exposure and anastomosis.

Vascular anastomosis is typically the most challenging procedure during laparoscopic or robotic surgery, and the quality of which strongly impacts post-transplant outcomes. Our group has been working on magnetic-assisted vascular anastomosis for more than 20 years, and have successfully utilized it in animals and clinical patients^5–9^. Previously, we have used combined magnetic devices for supra-IVC and infra-IVC, as well as portal vein anastomosis in six patients, and shorted the anhepatic time to 10.5 min^7^. In the current study, the native liver explanation was performed laparoscopically. In addition, we further simplified the procedure for magnetic-assisted portal vein anastomosis. Specifically, the donor vascular wall was everted on the magnet and ligated for fixation with the gap of the magnetic ring at 12 o’clock direction. For the recipient portal vein, the vascular wall was similarly everted on the other magnet with the gap at 12 o’clock, and subsequent attachment of the two magnets allows for a fast blood reperfusion after withdrawal of the ligation on the donor portal vein. Some skills should be addressed. First, during operation close to the magnets, special instruments, for example, forceps, needle holders, made of nonmagnetic titanium alloy are more appropriate. In addition, the gap of the C-shaped magnetic ring marks of the direction of portal vein for prevention of vascular distortion, and allows bloodletting after reperfusion. As such, continuous suturing always starts at the gaps. Moreover, the simplified magnetic anastomosis versus previous procedure further saves the time^7^, and makes the length of the portal vein adjustable before and even after attachment of the two magnets. In fact, by using fast magnetic portal vein anastomosis, the median anhepatic time was shortened to 25 min in the current study, which was significantly less than the previous laparoscopic/robotic procedures (43–240 min)(Table 2). Taken together, the simplified magnetic-assisted portal vein anastomosis is applicable for fast blood reperfusion during liver transplantation, and might promote the progress of minimally invasive liver transplantation.

Among the limitations, the current study has only successfully included eight patients in a single-center due to the initial experience. Future multi-institutional study with a larger sample size and comparable control groups are needed. In conclusion, LA-LT with full-sized graft and a midline incision is feasible, and utilization of magnetic-assisted portal vein anastomosis significantly shortens the anhepatic time and further simplify the procedure.

## Ethical approval

The study was approved by the institutional ethical committee of The First Affiliated Hospital of Xi’an Jiaotong University (XJTU1AF2015LSL-046).

## Consent

All cases suitable for LA-LT were discussed at a multidisciplinary team meeting, and informed consent were obtained from all the patients and their families.

## Source of funding

This study was supported by National Natural Science Foundation of China (30830099, 81470896, 81127005).

## Author contribution

Drs X.-F.Z. and Y.L.: had full access to all of the data in the study and take responsibility for the integrity of the data and the accuracy of the data analysis; Y.L., X.-M.L., X.-G.Z., Z.F., S.-P.W., J.-X.X., A.-H.S., X.-F.Z., and Y.L.: Concept and design; Y.L., X.-G.Z., S.-P.W., Z.F., J.-X.X., K.G., J.-J.H., and S.-N.L.: acquisition, analysis, or interpretation of data; Z.F. and X.-F.Z.: drafting of the manuscript; X.-F.Z. and Y.L.: critical revision of the manuscript for important intellectual content; Z.F. and X.-F.Z.: administrative, technical, or material support; X.-M.L., X.-F.Z., and Y.L.: supervision.

## Conflicts of interest disclosure

The authors declare that there are no conflicts of interest.

## Research registration unique identifying number (UIN)

This one has not been registered, but we have registered in Clinical trials for kinds of magnetic anastomosis techniques (NCT03774589, NCT03792048, etc.).

## Guarantor

Professors Yi Lyu and Xu-Feng Zhang.

## Data availability statement

The primary data could be accessed via contacting with the corresponding author.

## Provenance and peer review

Not commissioned, externally peer-reviewed.

## Supplementary Material
